# A national survey of physical activity after spinal cord injury

**DOI:** 10.1038/s41598-022-07927-5

**Published:** 2022-03-15

**Authors:** Jan Elaine Soriano, Jordan W. Squair, Jacquelyn J. Cragg, Jennifer Thompson, Rafael Sanguinetti, Bita Vaseghi, Carolyn A. Emery, Christopher Grant, Rebecca Charbonneau, Kelly A. Larkin-Kaiser, Aaron A. Phillips, Zeljko Dujic

**Affiliations:** 1 Departments of Physiology and Pharmacology, Cardiac Sciences & Clinical Neurosciences, Libin Cardiovascular Institute of Alberta, Hotchkiss Brain Institute, University of Calgary, 93 Heritage Medical Research Building, 3310 Hospital Dr NW, Calgary, AB T2N 4N1 Canada; 2 MD/PhD Training Program, Faculty of Medicine, University of British Columbia, Vancouver, Canada; 3 International Collaboration On Repair Discoveries (ICORD), University of British Columbia, Faculty of Pharmaceutical Sciences, 2405 Wesbrook Mall, Vancouver, BC V6T 1Z3 Canada; 4 Cumming School of Medicine, University of Calgary, 93 Heritage Medical Research Building, 3310 Hospital Dr NW, Calgary, AB T2N 4N1 Canada; 5 Sport Injury Prevention Research Centre, Faculty of Kinesiology and Departments of Pediatric and Community Health Sciences, Cumming School of Medicine, University of Calgary, 2500 University Dr NW, Calgary, AB T2N 1N4 Canada; 6 Division of Physical Medicine & Rehabilitation, Department of Clinical Neurosciences, Cumming School of Medicine, University of Calgary, Foothills Medical Centre, 1403 29th Street NW, Calgary, AB T2N 2T9 Canada; 7 Department of Integrative Physiology, School of Medicine, University of Split, Šoltanska 2, 21000 Split, Croatia; 8 University of Calgary, 78 Heritage Medical Research Building, 3310 Hospital Dr NW, Calgary, AB T2N 4N1 Canada

**Keywords:** Spinal cord diseases, Physiology

## Abstract

Physical activity is a powerful modifiable risk factor for disease and mortality. Physical activity levels in people with spinal cord injury (SCI) have not been quantified relative to uninjured individuals in a large population-based sample. We aimed to quantify and compare physical activity in people with and without SCI, and to examine the associations between physical activity, lifestyle, and socioeconomic factors. The 2010 Canadian Community Health Survey (n > 57,000) was used, which includes three measures that assess physical activity levels (i.e., leisure time activity frequency, leisure time activity intensity, and transportation time activity intensity). Bivariable and multivariable logistic regressions were performed and odds ratios (ORs) with corresponding 95% confidence intervals (CIs) were estimated. The odds of physical activity in people with SCI were 0.43 (95% CI 0.3–0.61), 0.53 (95% CI 0.36–0.75), and 0.42 (95% CI 0.28–0.61), across the three measures of physical activity, respectively. These differences persisted after adjustment for lifestyle, comorbidities, and socioeconomic factors. Physical activity is reduced in the SCI population compared with the general population. This knowledge is important to direct future research and guide the allocation of health care resources.

## Introduction

Low physical activity is a powerful modifiable risk factor for non-communicable diseases such as heart disease, stroke, and diabetes^1–3^. Spinal cord injury (SCI) is a devastating neurological condition leading to paralysis of skeletal muscle and autonomic cardiovascular dysfunction, both of which are profound barriers to being physically active^4^. People with SCI have higher rates of heart disease, stroke, and diabetes^2,3^. Increasing physical activity levels may be a powerful clinical intervention for preventing these conditions^1^. Physical activity, exercise, and rehabilitation are health care priorities for people with SCI^5^. Moreover, increased participation in leisure-time physical activity levels has a positive association with increased quality of life in people with SCI^6,7^. However, a previous study showed that approximately half of people with SCI report no leisure-time physical activity, and, it is not clear if this is a similar rate as those reported in non-SCI populations^8,9^. A key step in understanding the potential of increasing leisure-time physical activity on a large scale is to understand the population-level physical activity levels of people with SCI compared to the general population.

Various lifestyle and socioeconomic factors are associated with physical activity in the general population, including income, education level, gender, diet, cigarette smoking, and alcohol consumption^10–15^. Due to different lifestyles and socioeconomic profiles in the SCI population, the association between these factors and physical activity may be unique in people with SCI^16–21^. We do not understand the association of various lifestyle and socioeconomic factors with physical activity levels in the SCI population.

We aimed to compare the physical activity level of individuals with and without SCI on a population scale. We also aimed to understand the relationship between lifestyle and socioeconomic factors with physical activity levels in the SCI population. This knowledge is important to direct future research, and potential interventions and educational strategies for people with SCI, to ultimately reduce the development of risk factors that impact long-term health.

## Results

### Overall sample characteristics

The CCHS was completed by just over 57,000 individuals, of whom 330 self-reported SCI (Table 1). Each physical activity measure of the CCHS was completed by the following number of participants: leisure time activity frequency (n = 57,487); leisure time activity intensity (n = 57,497); transportation time activity intensity (n = 57,096). A similar proportion of males and females completed the survey (Table 1). The overall sample included a similar number of females (49.8%) and males (50.2%), with a median age of 50 to 59 years and a mean body mass index (BMI) of 26 kg/m^2^ (Table 1). Sample sizes differed following adjustments to confounders (Table 2).

**Table 1:** Characteristics of the population-based survey by physical activity status.

Variable		Frequency of all leisure time physical activity lasting over 15 min (PACDFR)			Leisure time physical activity index (PACDPAI)			Transportation and leisure time physical activity index (PACDLTI)		
		Total sample (n = 57,487)	Physical activity (n = 46,943)	No physical activity (n = 10,544)	Total sample (n = 57,497)	Physical activity (n = 30,358)	No physical activity (n = 27,139)	Total sample (n = 57,096)	Physical activity (n = 31,054)	No physical activity (n = 26,042)
**Spinal cord injury**										
Yes		330 (0.47)	198 (60.8)	132 (39.2)	330 (0.47)	116 (35.5)	214 (64.5)	326 (0.44)	114 (32.0)	212 (68.0)
No		57,157 (99.5)	46,745 (81.8)	10,412 (18.2)	57,167 (99.5)	30,242 (52.8)	26,925 (47.3)	56,770 (99.6)	30,940 (55.0)	25,830 (45.0)
**Sex**										
Male	SCI	202 (0.35)	125 (0.27)	77 (0.73)	202 (0.35)	73 (0.24)	129 (0.48)	199 (0.35)	71 (0.23)	128 (0.49)
	No SCI	26,275 (45.71)	21,854 (46.55)	4,421 (41.92)	26,285 (45.72)	14,586 (48.05)	11,699 (43.11)	26,067 (45.65)	14,924 (48.06)	11,143 (42.79)
Female	SCI	128 (0.22)	73 (0.16)	55 (0.52)	128 (0.22)	43 (0.14)	85 (0.31)	127 (0.22)	43 (0.14)	84 (0.32)
	No SCI	30,882 (53.72)	24,891 (53.02)	5,991 (56.82)	30,882 (53.71)	15,656 (51.57)	15,226 (56.10)	30,703 (53.77)	16,016 (51.57)	14,687 (56.40)
Median age category (years)	SCI	55–59	55–59	52–56	55–59	55–59	55–59	55–59	55–59	55–59
	No SCI	50–54	45–49	55–59	50–54	45–49	50–54	50–54	45–49	55–59
Median BMI (kg/m^2^)	SCI	26	26	27	26	25	27	26	25	27
	No SCI	25	25	26	25	25	26	25	25	26

*SCI* spinal cord injury, *BMI* body mass index.

**Table 2:** Characteristics of the population-based survey by physical activity status following adjustments to confounders.

**Variable**		Frequency of all leisure time physical activity lasting over 15 min (PACDFR)			Leisure time physical activity index (PACDPAI)			Transportation and leisure time physical activity index (PACDLTI)		
		Total sample (n = 35,666)*	Active (n = 30,385)	Inactive (n = 5,281)	Total sample (n = 35,675)*	Active (n = 20,136)	Inactive (n = 15,539 )	Total sample (n = 35,437)*	Active (n = 20,643)	Inactive (n = 14,794)
**Smoking**										
Yes	SCI	126 (0.35)	72 (0.24)	54 (1.02)	74 (0.21)	46 (0.23)	80 (0.51)	125 (0.35)	45 (0.22)	80 (0.54)
	No SCI	16,296 (45.69)	13,390 (44.07)	2906 (55.03)	16,350 (45.83)	8505 (42.24)	7793 (50.15)	16,222 (45.78)	8694 (42.12)	7528 (50.88)
No	SCI	55 (0.15)	37 (0.12)	18 (0.34)	55 (0.15)	28 (0.14)	27 (0.17)	54 (0.15)	27 (0.13)	27 (0.18)
	No SCI	19,189 (53.80)	16,886 (55.57)	2303 (43.61)	19,196 (53.81)	11,557 (57.39)	7639 (49.16)	19,036 (53.72)	11,877 (57.53)	7159 (48.39)
**Hypertension**										
Yes	SCI	22 (0.06)	9 (0.03)	13 (0.25)	22 (0.06)	8 (0.04)	14 (0.09)	22 (0.06)	8 (0.04)	14 (0.09)
	No SCI	2311 (6.48)	1854 (6.10)	457 (8.65)	2311 (6.48)	1199 (5.95)	1112 (7.16)	2304 (6.50)	1219 (5.91)	1085 (7.33)
No	SCI	159 (0.45)	100 (0.33)	59 (1.12)	159 (0.45)	66 (0.33)	93 (0.60)	157 (0.44)	64 (0.31)	93 (0.63)
	No SCI	33,174 (93.01)	28,422 (9.35)	4752 (89.99)	33,183 (93.01)	18,863 (93.68)	14,320 (92.16)	32,954 (92.99)	19,352 (93.75)	13,602 (0.09)
**Alcohol**										
High	SCI	125 (0.35)	78 (0.26)	47 (0.89)	125 (0.35)	55 (0.27)	70 (0.45)	124 (0.35)	54 (0.26)	70 (0.47)
	No SCI	28,128 (78.86)	24,392 (80.28)	3736 (70.74)	28,136 (78.87)	16,183 (80.37)	11,953 (76.92)	27,963 (78.91)	16,608 (80.45)	11,355 (76.75)
Low	SCI	56 (0.16)	31 (0.10)	25 (0.47)	56 (0.16)	19 (0.09)	37 (0.24)	55 (0.16)	18 (0.09)	37 (0.25)
	No SCI	7357 (20.63)	5884 (19.36)	1473 (27.89)	7358 (20.63)	3879 (19.26)	3479 (22.39)	7295 (20.59)	3963 (19.20)	3332 (22.52)
**Fruits and vegetables**										
High	SCI	84 (0.23)	55 (0.18)	29 (0.55)	84 (0.24)	43 (0.21)	41 (0.26)	83 (0.23)	42 (0.20)	41 (0.28)
	No SCI	18,037 (50.57)	16,156 (53.17)	1881 (35.62)	18,039 (50.56)	11,747 (58.34)	6292 (40.49)	17,891 (50.49)	11,960 (57.94)	5931 (40.09)
Low	SCI	97 (0.27)	54 (0.18)	43 (0.81)	97 (0.27)	31 (0.15)	66 (0.42)	96 (0.27)	30 (0.15)	66 (0.45)
	No SCI	17,448 (48.92)	14,120 (46.47)	3328 (63.02)	17,455 (48.93)	8315 (41.29)	9140 (58.82)	17,367 (49.01)	8611 (41.71)	8756 (59.19)
**Income**										
High	SCI	90 (0.25)	59 (0.19)	31 (0.59)	90 (0.25)	39 (0.19)	51 (0.33)	89 (0.25)	38 (0.18)	51 (0.34)
	No SCI	25,455 (71.37)	22,574 (74.29)	2881 (54.55)	25,463 (71.37)	15,315 (76.06)	10,148 (65.31)	25,277 (71.33)	15,670 (75.91)	9607 (64.94)
Low	SCI	91 (0.25)	50 (0.16)	41 (0.78)	91 (0.26)	35 (0.17)	56 (0.36)	90 (0.25)	34 (0.16)	56 (0.38)
	No SCI	10,030 (28.12)	7702 (25.35)	2328 (44.08)	10,031 (28.12)	4747 (23.57)	5284 (34.00)	9981 (28.17)	4901 (23.74)	5080 (34.34)
**Education**										
High	SCI	128 (0.36)	81 (0.27)	47 (0.89)	128 (0.36)	55 (0.27)	73 (0.47)	126 (0.36)	53 (0.26)	73 (0.49)
	No SCI	28,691 (80.44)	25,172 (82.84)	3519 (66.63)	28,699 (80.45)	16,980 (84.33)	11,719 (75.42)	28,502 (80.43)	17,438 (84.47)	11,064 (74.79)
Low	SCI	53 (0.15)	28 (0.09)	25 (0.47)	53 (0.15)	19 (0.09)	34 (0.22)	53 (0.15)	19 (0.09)	34 (0.23)
	No SCI	6794 (19.05)	5104 (16.80)	1690 (32.00)	6795 (19.05)	3082 (15.31)	3713 (23.89)	6756 (19.06)	3133 (15.18)	3623 (24.49)
**Self-perceived mental health**										
Good	SCI	37 (0.10)	19 (0.06)	18 (0.34)	37 (0.10)	15 (0.07)	22 (0.14)	37 (0.10)	15 (0.07)	22 (0.15)
	No SCI	1799 (5.04)	1298 (4.27)	501 (9.49)	1799 (5.04)	741 (3.68)	1058 (6.81)	1789 (5.04)	771 (3.73)	1018 (6.88)
Poor	SCI	144 (0.40)	90 (0.30)	54 (1.02)	144 (0.40)	59 (0.29)	85 (0.55)	142 (0.40)	57 (0.28)	85 (0.57)
	No SCI	33,686 (94.45)	28,978 (95.37)	4708 (89.15)	33,695 (94.45)	19,321 (95.95)	14,374 (92.50)	33,469 (94.45)	19,800 (95.92)	13,669 (92.40)
**Stress**										
High	SCI	132 (0.37)	76 (0.25)	56 (1.06)	132 (0.37)	50 (0.25)	82 (0.53)	130 (0.37)	48 (0.23)	82 (0.55)
	No SCI	22,039 (61.79)	18,754 (61.72)	3285 (62.20)	22,044 (61.79)	7917 (39.32)	14,127 (90.91)	21,907 (61.82)	12,479 (60.45)	9428 (63.73)
Low	SCI	49 (0.14)	33 (0.11)	16 (0.30)	49 (0.14)	24 (0.12)	25 (0.16)	49 (0.14)	24 (0.12)	25 (0.17)
	No SCI	13,446 (37.70)	11,522 (37.92)	1924 (36.43)	13,450 (37.70)	12,145 (60.31)	1305 (8.40)	13,351 (37.68)	8092 (39.20)	5259 (35.55)
**Migraines**										
Yes	SCI	44 (0.12)	23 (0.08)	21 (0.40)	44 (0.12)	16 (0.08)	28 (0.18)	43 (0.12)	15 (0.07)	28 (0.19)
	No SCI	3574 (10.02)	3001 (9.88)	573 (10.85)	3574 (10.02)	1907 (9.47)	1667 (10.73)	3548 (9.96)	1954 (9.47)	1594 (10.77)
No	SCI	137 (0.38)	86 (0.28)	51 (0.97)	137 (0.38)	58 (0.29)	79 (0.51)	136 (0.38)	57 (0.28)	79 (0.53)
	No SCI	31,911 (89.47)	27,275 (89.76)	4636 (87.77)	31,920 (89.47)	18,155 (90.16)	13,765 (88.58)	31,710 (89.48)	18,617 (90.19)	13,093 (88.50)
**Mood disorder**										
Yes	SCI	45 (0.13)	24 (0.08)	21 (0.40)	45 (0.13)	19 (0.94)	26 (0.17)	44 (0.12)	18 (0.09)	26 (0.18)
	No SCI	2516 (7.54)	1955 (6.43)	561 (10.62)	2516 (7.05)	1125 (5.59)	1391 (8.95)	2502 (7.06)	1157 (5.60)	1345 (9.09)
No	SCI	136 (0.38)	85 (0.28)	51 (0.97)	136 (0.38)	55 (0.27)	81 (0.52)	135 (0.38)	54 (0.26)	81 (0.55)
	No SCI	32,969 (92.44)	28,321 (93.21)	4648 (88.01)	32,978 (92.44)	18,937 (94.05)	14,041 (90.36)	32,756 (92.43)	19,414 (94.05)	13,342 (90.19)
**Anxiety disorder**										
Yes	SCI	29 (0.08)	16 (0.05)	13 (0.25)	29 (0.08)	13 (0.06)	16 (0.10)	29 (0.08)	13 (0.060	16 (0.11)
	No SCI	1857 (5.21)	1466 (4.82)	391 (7.40)	1857 (5.21)	915 (4.54)	942 (6.06)	1847 (5.21)	940 (4.55)	907 (6.13)
No	SCI	152 (0.43)	93 (0.31)	59 (1.12)	152 (0.43)	61 (0.30)	91 (0.59)	150 (0.42)	59 (0.29)	91 (0.62)
	No SCI	33,628 (94.29)	28,810 (94.82)	4818 (91.23)	33,637 (94.29)	19,147 (0.95)	14,490 (93.25)	33,411 (94.28)	19,631 (95.10)	13,780 93.15)

Data are sample sizes (percentages). Percentages are probability-weighted.

*SCI* spinal cord injury, *BMI* body mass index.

*The total sample size of the participants who responded to all the confounding variables.

### Individuals with SCI are at greater risk of physical inactivity compared to able-bodied individuals

In individuals with SCI relative to able-bodied individuals, the unadjusted OR for leisure time activity frequency was 0.35 (95% CI 0.27–0.44) (Table 3). After adjusting for age, sex, and BMI the OR slightly increased to 0.39 (95% CI 0.30–0.50) (Table 3). Additionally, even after inclusion of all these variables, the results remained significant (adjusted OR 0.43; 95% CI 0.30–0.61; ROC 0.69). In individuals with SCI relative to able-bodied individuals, the unadjusted OR for leisure time activity intensity was 0.49 (95% CI 0.38–0.63) (Table 3). After adjusting for age, sex, and BMI the OR slightly increased to 0.55 (95% CI 0.43–0.71) (Table 3). Additionally, even after inclusion of all these variables, the results remained significant (adjusted OR 0.53; 95% CI 0.36–0.75; ROC 0.64). In individuals with SCI relative to able-bodied individuals, the unadjusted OR for transportation time activity intensity was 0.38 (95% CI 0.29–0.50) (Table 3). After adjusting for age, sex, and BMI the OR slightly increased to 0.43 (95% CI 0.33–0.57). Additionally, even after inclusion of all these variables, the results remained significant (adjusted OR 0.42; 95% CI 0.28–0.61; ROC 0.65) (Table 3). In other words, all three elements demonstrated converging results that those with SCI participate in less physical activity compared to people without SCI. These results remained significant after adjusting for age, sex, and BMI, as well as including all potential confounders (Tables 4 and 5).

**Table 3:** Odds ratios for association between spinal cord injury and physical activity (probability-weighted).

	Frequency of all leisure time physical activity lasting over 15 min			Leisure time physical activity index			Transportation and leisure time physical activity index		
	OR (95% CI)	aOR (95% CI)	aOR2 (95% CI)	OR (95% CI)	aOR (95% CI)	aOR2 (95% CI)	OR (95% CI)	aOR (95% CI)	aOR2 (95% CI)
Spinal cord injury	0.35* (0.27, 0.44)	0.39* (0.30, 0.50)	0.43* (0.30, 0.61)	0.49* (0.38, 0.63)	0.55* (0.43, 0.71)	0.53* (0.36, 0.75)	0.38* (0.29, 0.50)	0.43* (0.33, 0.57)	0.42* (0.28, 0.61)
No spinal cord injury^a^	1.0	1.0	1.0	1.0	1.0	1.0	1.0	1.0	1.0

*CI* confidence interval, *OR* odds ratio.

^a^Reference category. *Statistically significant p < 0.05.

**Table 4:** Spinal cord injury status, sex, age, body mass index and comorbidities.

Variable name	CCHS code	Question	Response values	Combined response values (*reference category)	Excluded responses
Spinal cord injury status	NEUDSIR	“Do you have a neurological condition caused by a spinal cord injury?”	1 = “Yes” 2 = “No” (reference category)	(*2)	9 = “At least one required question was not answered (don’t know, refusal, not stated)”
Sex	DHH_SEX	Is [respondent name] male or female?	1 = “Male” 2 = “Female”	(*2)	N/A
Age	DHHGAGE	Questions are described in detail here^22^	1 = “Age between 12 and 14” 2 = “Age between 15 and 17” 3 = “Age between 18 and 19” 4 = “Age between 20 and 24” 5 = “Age between 25 and 29” 6 = “Age between 30 and 34” 7 = “Age between 35 and 39” 8 = “Age between 40 and 44” 9 = “Age between 45 and 49” 10 = “Age between 50 and 54” 11 = “Age between 55 and 59” 12 = “Age between 60 and 64” 13 = “Age between 65 and 69” 14 = “Age between 70 and 74” 15 = “Age between 75 and 79” 16 = “Age 80 and older”	N/A	N/A
Body mass index	HWTGBMI	Questions are described in detail here^22^	14 = Minimum 58 = Maximum Body mass index was categorized into the following groups: normal (18.5–25), overweight (25–30), obese class I (30–35), obese class II (35–40), obese class III (> 40)	1—less than the median HWTGBMI of all respondents 2—greater or equal to the median HWTGBMI of all respondents (*2)	N/A = Height and/or weight was not given; Respondent less than 20 or more than 64 years old; or Respondent is pregnant
Hypertension	CCC_072	“Have you ever been diagnosed with high blood pressure?”	1 = “Yes” 2 = “No”	(*1)	N/A
Migraine	CCC_081	“Do you have migraine headaches?”	1 = “Yes” 2 = “No”	(*1)	N/A
Mood disorders	CCC_280	“Do you have a mood disorder such as depression, bipolar disorder, mania or dysthymia?”	1 = “Yes” 2 = “No”	(*1)	N/A
Anxiety disorders	CCC_290	“Do you have an anxiety disorder such as phobia, obsessive–compulsive disorder or a panic disorder?”	1 = “Yes” 2 = “No”	(*1)	N/A

*CCHS* Canadian Community Health Survey.

**Table 5:** Lifestyle and socioeconomic factors.

Variable name	CCHS code	Question	Response values	Combined response values (*reference category)	Excluded responses
Smoking	SMKDSTY (derived variable) SMK_01A, SMK_01B, SMK_202 and SMK_05D	Questions are described in detail here^22^	1—“Daily smoker” 2—“Occasional smoker (former daily smoker)” 3—Occasional smoker (never a daily smoker or has smoked less than 100 cigarettes lifetime) 4—“Former daily smoker (non-smoker now)” 5—“Former occasional smoker (at least 1 whole cigarette, non-smoker now)” 6—“Never smoked (a whole cigarette)”	1—1, 2, 3, 4 2—5, 6 (*1)	99—“At least one required question was not answered (don’t know, refusal, not stated)”
Alcohol consumption	ALCDTTM (derived variable) ALC_1 ALC_2	Questions are described in detail here^22^	1—“Regular drinker” 2—“Occasional drinker” 3—“Did not drink in the last 12 months”	1—1, 2 2—3 (*1)	9—“At least one required question was not answered (don’t know, refusal, not stated)”
Fruit and vegetable consumption	FVCDTOT	Questions are described in detail here^22^	0 = Minimum 120 = Maximum	1—less than the median FVCDTOT of all respondents 2—greater or equal to the median FVCDTOT of all respondents (*1)	999.99—“At least one required question was not answered (don't know, refusal, not stated)”
Self-perceived mental health and life stress	GEN_02B	“In general, would you say your mental health is:	1—…excellent?” 2—…very?” 3—good?” 4—…fair?” 5—…poor?”	1—1, 2, 3 2—4, 5 (*1)	N/A
	GEN_07	“Thinking about the amount of stress in your life, would you say that most days are:	1—…not at all stressful? 2—…not very stressful? 3—…a bit stressful? 4—…quite a bit stressful? 5—…extremely stressful?”	1—1, 2, 3 2—4, 5 (*1)	N/A
Total household income	INCGHH INCDHH INC_5A INC_5B INC_5C	Questions are described in detail here^22^	1—“No income or less than 20,000” 2—“$20,000 to $39,999” 3—“$40,000 to $59,999” 4—“$60,000 to $79,999” 5—“$80,000 or more”	1—1 2—2, 3, 4, 5 (*1)	9—“Required question was not answered (don’t know, refusal, not stated)”
Educational level	EDUDH04 EDUDR04 EDU_1, EDU_2, EDU_3 and EDU_4	Questions are described in detail here^22^	1—“Less than secondary school graduation” 2—“Secondary school graduation, no post-secondary education” 3—“Some post-secondary education” 4—“Post-secondary degree/diploma”	1—1, 2 2—3, 4 (*1)	9—“At least one required question was not answered (don’t know, refusal, not stated)”

*CCHS* Canadian Community Health Survey.

#### The interaction between physical activity with lifestyle and socioeconomic factors in people with and without SCI

**Figure 1:**
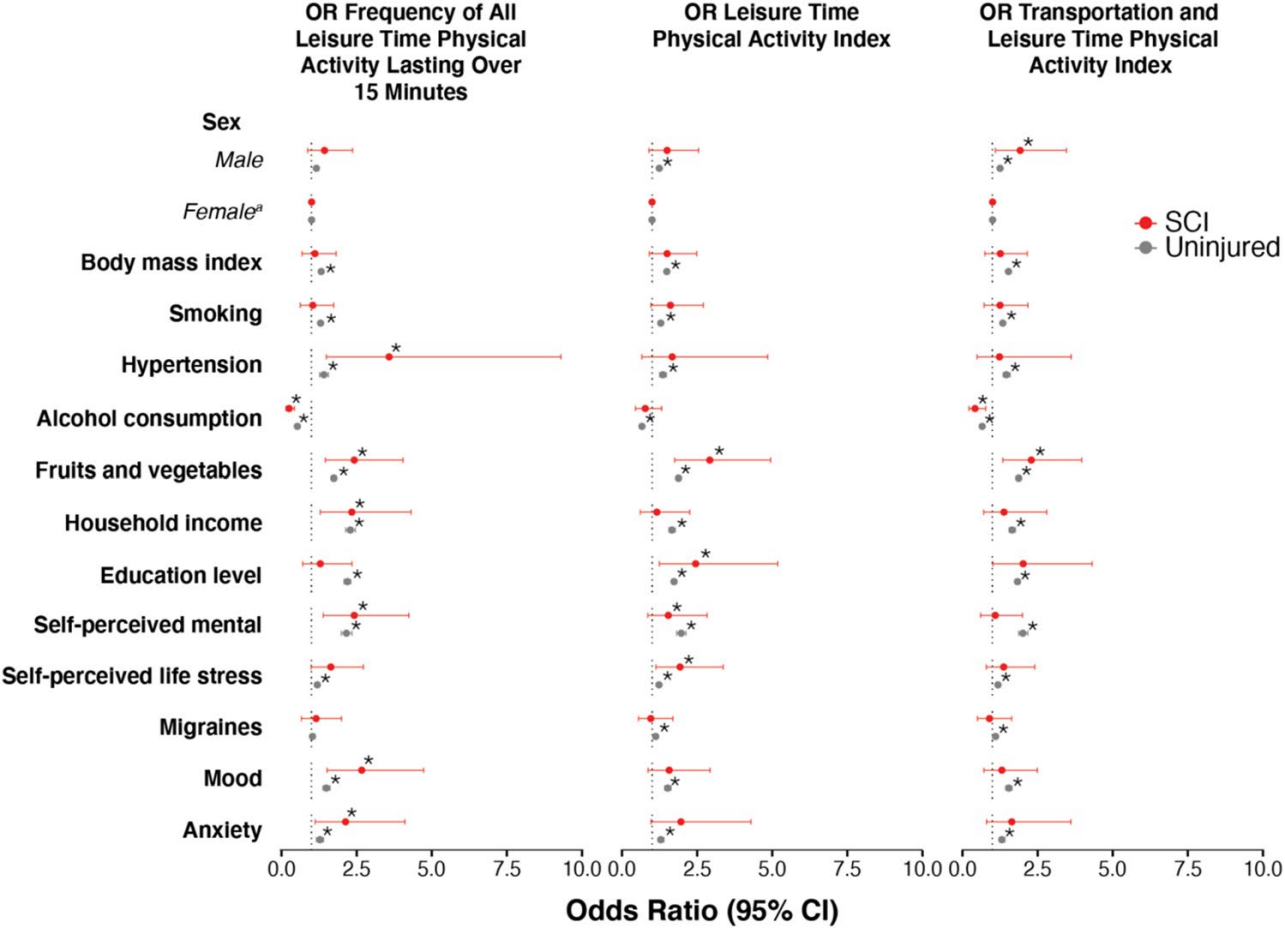
Lifestyle and socioeconomic factors. Odds ratio and 95% confidence intervals for the association between physical activity levels and lifestyle and socioeconomic variables. ORs derived from logistic regression models. *SCI* spinal cord injury, *CI* confidence interval, *OR* odds ratio.

In people with SCI, there was no association between physical activity level and sex, body mass index, migraines, or smoking. This is in contrast to people without SCI where male sex, lower body mass index, not smoking, and absence of migraines were associated with increased physical activity levels (Fig. 1). In both those with and without SCI, there was an association between increased physical activity levels and not being diagnosed with hypertension, eating more fruits and vegetables, greater household income, greater education levels, better mental health, lower stress levels, reduced likelihood of mood disorders, and reduced anxiety (Fig. 1). For details on lifestyle and socioeconomic factors, see Tables 4 and 5.

## Discussion

On a population-scale, physical activity levels are reduced in people with SCI compared to uninjured individuals. This result persisted after adjustment for age, sex, BMI, as well as numerous lifestyle and socio-economic factors. Lower-income, education, and fruit and vegetable consumption and increased alcohol use were associated with reduced physical activity in the SCI population. As such, specific socio-economic groups within the SCI population may benefit most from physical activity promotion activities.

### Physical activity levels are reduced in those with SCI

Reduced physical activity in people with SCI is likely the result of numerous physical factors, including loss of skeletal muscle control, reduced skeletal muscle mass, reduced cardiovascular reserve, as well as environmental and social barriers^23,24^. Moreover, other psychological and behavioural barriers to engagement in physical activity include a lack of knowledge, community support, beliefs in ability, coping, conflicting goals for rehabilitation and limited access to disability-related experts and accessible rehabilitation infrastructures^25–27^. Reduced physical activity plausibly contributes to widespread cardiometabolic disorders after SCI, increased cardiovascular disease and diabetes risk, and a range of physical, emotional, and mental health issues that affect people with SCI^1,3,28–30^. As such, it is essential that targeted physical activity interventions for individuals with SCI integrate physical, psychological and behavioural based approaches to support effective uptake of interventional strategies and ensure the mitigation of these risk factors. Furthermore, strategies to increase physical activity may be more successful if they are integrated into adapted activities and/or those that directly align with an individual's goals (i.e., gardening, walking a dog, cycling, resistance training, yoga etc.)^31^.

Previous research has shown that approximately half of people with SCI report no leisure-time physical activity. However, it is not clear if this proportion differs from non-SCI populations, who also report very high rates of physical inactivity^8,9^. Another study compared physical activity levels in 40 people with SCI to age-matched uninjured controls, showing that individuals with SCI have reduced durations of dynamic activity one year after discharge from rehabilitation^32^. Our data provide additional support for these findings on a population-scale with control individuals.

There is a unique profile of lifestyle factors associated with physical activity levels within the SCI population. Within the SCI population there is no association between male sex, reduced body mass index, less likelihood of migraines, and not smoking with physical activity levels. This may be due to a variety of factors that are outside the scope of this study to quantitatively evaluate. Some of these may include the interaction between gender and self-efficacy before and after SCI^33–35^, as well as the interaction between body mass index and severity of disability after SCI^36^.

In the present data, greater physical activity was associated with an improved lifestyle. Greater physical activity levels in people with SCI were also associated with a reduced likelihood of being diagnosed with hypertension, anxiety and mood disorders, better mental health, and lower self-reported stress (Fig. 1). Greater household income and education, as well as lower alcohol consumption and eating more fruits and vegetables, were also factors associated with increased physical activity levels. Lower education and household income in the general population have also been associated with low physical activity levels^37^. It is reasonable to expect that these factors, in combination with the consequences of SCI, may be further exaggerated in the SCI population. These specific demographic groups within the SCI population (those with lower education and household incomes), should therefore be precision-targeted to understand exercise barriers, potential education programs needed and ultimately establish effective interventional strategies that promote physical activity. For example, previous public health interventions have promoted active transportation involving human energy to mobilize and travel. These programs have demonstrated success as they have been established by creating safe environments (e.g., improving community landscape, adding more sidewalks, longer pedestrian crossing times on signal lights) for the general population including pedestrians, bikers, as well as wheelchair users^38,39^. Interventional programs such as these provide equal opportunities for individuals with varying socioeconomic backgrounds to access transportation options and provide an example of an effective strategy that integrated the physical, behavioural, and social needs of multiple demographics to encourage uptake of a desired outcome for a broad population.

### Limitations

A primary strength of this study was the use of the CCHS databases, as the sample selected is designed to be representative of the Canadian population (~ 34 million adults), and therefore the data is considered highly generalizable^40^. Furthermore, the ~ 330 respondents with SCI represent ~ 0.4% of the population of Canadians living with SCI^41^. It is also unlikely that our results suffered from response bias as is expected in a single topic survey when respondents would potentially aim to answer questions in the style that the interviewers prefer. Although self-reported physical activity levels in people with SCI are subjective, they do relate to objective physical activity measures^42^. However, the specific questions asked in the CCHS have not been assessed in comparison to objective measures. The CCHS data are derived from a cross-sectional study design and it is therefore not possible to determine the causality between variables. It is possible that misclassification occurred in terms of level and severity of injury. This would be most likely for individuals with less severe SCI, who would be expected to participate in physical activity more frequently than those with higher more complete SCI^43^. Therefore, including individuals with lower severity scores may result in an underestimation of the reported effect size.

## Conclusions

On a population-level, physical activity levels in people with SCI are reduced, even after controlling for lifestyle and socioeconomic factors. Specific socio-economic groups within the SCI population, such as those in lower socioeconomic demographics, may benefit most from physical activity promotion activities.

## Methods

### Standard protocol approvals, registrations, and patient consents

All research was performed in accordance with relevant guidelines/regulations. The Tri-Council Policy Statement on Ethical Conduct for Research Involving Humans (TCPS2) states that use of publicly available, de-identified information, such as Statistics Canada data (i.e., the Canadian Community Health Survey), is exempt from review by institutional research ethics boards. This policy was confirmed by the University of Calgary Conjoint Health Research Ethics Board.

### Data source

To evaluate physical activity in individuals with SCI compared to individuals without SCI, the data were accessed through the 2010 component of the Canadian Community Health Survey (CCHS). The CCHS is a comprehensive national survey conducted by Statistics Canada. The survey is voluntary and conducted on individuals aged 12 years and older, who reside in households across all Canadian provinces and territories^44^. Individuals living on reserves or Crown lands, full-time members of the Canadian armed forces, and those living in institutions (i.e., prisons, hospitals, universities) are excluded from the survey.

### Exposure and outcome definitions

#### SCI status

SCI status was obtained with the question: “Do you have a neurological condition caused by a spinal cord injury?” During the survey, individuals were given the following reminder: “Remember, we’re interested in conditions diagnosed by a health professional.” Only those with valid responses for the primary explanatory variable and outcome variables were included in the analysis. Non-respondents (those in the categories of “don’t know,” “refusal,” and “not stated”) were excluded. The questions are described in detail here^22,45^.

#### Frequency of all leisure time physical activity lasting over 15 min

To capture “leisure time activity frequency”, we used the PACDFR variable from the CCHS. This variable classifies respondents as having “regular practice of leisure time activities”, “occasional practice of leisure time activities” and “infrequent practice of leisure time activities” lasting over 15 min based on the monthly frequency of physical activity reported for a three-month period. The questions are described in detail here^22,45^. Responses for PACDFR were binarized (Table 6).

**Table 6:** Measures of physical activity levels.

Variable name	CCHS code	Question	Response values	Combined response values (*reference category)	Excluded responses
**Physical activity levels**					
Frequency of all leisure time physical activity lasting over 15 min	PACDFR	Questions are described in detail here^22^	1 = “Regular practice of leisure time activities” 2 = “Occasional practice of leisure time activities” 3 = “Infrequent practice of leisure time activities”	1—1, 2 2—3 (*2)	9 = Required question was not answered (don’t know, NS refusal, not stated)
Leisure time physical activity index	PACDPAI	Questions are described in detail here^22^	1 = “Active” 2 = “Moderately active” 3 = “Inactive”	1—1, 2 2—3 (*2)	9 = At least one required question was not answered (don’t know, refusal, not stated)
Transportation and leisure time physical activity index	PACDLTI	Questions are described in detail here^4^	1 = “Active” 2 = “Moderately active” 3 = “Inactive”	1—1, 2 2—3 (*2)	9 = At least one required question was not answered (don’t know, refusal, not stated)

*CCHS* Canadian Community Health Survey.

#### Leisure time physical activity index

To capture “leisure time activity intensity”, we used the PACDPAI variable from the CCHS.

This variable categorizes respondents as being "active", "moderately active", or "inactive" in their leisure time based on the reported total daily Energy Expenditure values (kcal/kg/day) during the past three months. The questions are described in detail here^22,45^. Responses for PACDPAI were binarized (Table 6).

#### Transportation and leisure time physical activity index

To capture “transportation time activity intensity”, we used the PACDLTI variable from the CCHS. This variable categorizes respondents as being "active", "moderately active", or "inactive" in their transportation and leisure time based on the average daily energy expended (kcal/kg/day) during transportation and leisure-time physical activities by the respondent in the past three months. The questions are described in detail here^22,45^. Responses for PACDLTI were binarized (Table 6).

For details on physical activity level variables, see Table 6.

### Comorbidities

Previous diagnosis of hypertension (CCC_072) was obtained with the following question: “Have you ever been diagnosed with high blood pressure?” Migraine status (CCC_081) was obtained with the following question: “Do you have migraine headaches?” Previous diagnosis of mood disorders (CCC_280) was obtained with the following question: “Do you have a mood disorder such as depression, bipolar disorder, mania or dysthymia?” Previous diagnosis of anxiety disorders (CCC_290) was obtained with the following question: “Do you have an anxiety disorder such as phobia, obsessive–compulsive disorder or a panic disorder?” An individual could provide a “Yes” or “No” answer to the aforementioned questions. The questions are described in detail here^22,45^.

### Lifestyle and socioeconomic factors

Smoking status (SMKDSTY) indicates the type of smoker the respondent is. The questions are described in detail here^22,45^. The possible answers for SMKDSTY are “Daily smoker”, “Occasional smoker (former daily smoker)”, “Occasional smoker (never a daily smoker or has smoked less than 100 cigarettes lifetime)”, “Former daily smoker (non-smoker now)”, “Former occasional smoker (at least 1 whole cigarette, non-smoker now)”, “Never smoked (a whole cigarette)”, or “At least one required question was not answered (don’t know, refusal, not stated)”. Responses for SMKDSTY were binarized (Table 5).

Alcohol consumption status (ALCDTTM) indicates the type of drinker the respondent is for the past 12 months. The questions are described in detail here^22,45^. The possible answers for ALCDTTM are “Regular drinker”, “Occasional drinker”, “Did not drink in the last 12 months”, or “At least one required question was not answered (don’t know, refusal, not stated)”. Responses for ALCDTTM were binarized (Table 5).

Fruit and vegetable consumption (FVCGTOT) was obtained based on the derived variable FVCDTOT (indicates the total number of times per day the respondent consumes fruits and vegetables [i.e., fruit juice, fruits, green salad, potatoes, and carrots]). The questions are described in detail here^22,45^. The possible answers for FVCGTOT are “Eats fruits and vegetables less than 5 times per day”, “Eats fruits and vegetables between 5 and 10 times per day”, Eats fruits and vegetables more than 10 times per day”, or “At least one required question was not answered (don't know, refusal, not stated)”. Responses for FVCGTOT were binarized (Table 5).

Self-perceived mental health (GEN_02B) was obtained with the question: “In general, would you say your mental health is: …excellent?, …very good?, …good?, …fair?, …poor? ”, or “At least one required question was not answered (don't know, refusal, not stated)”. Self-perceived life stress (GEN_07) was obtained with the question: “Thinking about the amount of stress in your life, would you say that most days are: …not at all stressful?, …not very stressful?, …a bit stressful?, …quite a bit stressful?, or …extremely stressful?”, or “At least one required question was not answered (don't know, refusal, not stated)”. Responses for GEN_02B and GEN_07 were binarized (Table 5).

Total household income (INCGHH) is based on INCDHH (INC_5A, INC_5B and INC_5C). The questions are described in detail here^22,45^. The possible answers for INCDHH are “No income or less than 20,000”, “$20,000 to $39,999”, “$40,000 to $59,999”, “$60,000 to $79,999”, “$80,000 or more”, or “Required question was not answered (don’t know, refusal, not stated)”. Responses for INCGHH were binarized (Table 5).

The highest level of education attained within the household (EDUDH04) is based on the highest level of education for each member of the household (EDUDR04). The questions are described in detail here^22,45^. The possible answers are “Less than secondary school graduation”, “Secondary school graduation, no post-secondary education”, “Some post-secondary education”, “Post-secondary degree/diploma”, or “At least one required question was not answered (don’t know, refusal, not stated)”. Responses for EDUDH04 were binarized (Table 5).

### Statistical analysis

Logistic regression models were obtained separately for the binary outcome physical activity levels with SCI as the main explanatory variable, and with lifestyle and socio-economic factors as the main explanatory variable. Models were probability weighted to account for the clustering and stratification sampling design used by the CCHS (as previously reported)^2,46^. Separate logistic regression models were generated for the physical activity outcomes using the ‘glm’ (generalized linear model) function with the family argument set to ‘binomial()’ from the R Statistical Software package ‘stats’. R (R Core Team, 2017) was used for all statistical analyses. Using the logistic models, unadjusted and adjusted odds ratios (ORs) with 95% confidence intervals are presented. Goodness of fit for the full model was assessed using a receiver-operating curve (ROC). The ORs were then adjusted for potential confounders using multivariable logistic regression. In the multivariable model, age, sex, and body mass index were input as additional explanatory variables to calculate the adjusted model (AOR). The sensitivity analysis included the lifestyle and socioeconomic variables described above. A fully adjusted model (AOR2) including all these potential explanatory variables is then presented. Statistical significance was defined as a p-value ≤ 0.05. Data are presented in accordance with the STROBE guidelines of reporting^47^.

## Data Availability

The data that support the findings of this study are publicly available from Statistics Canada.
